# Relationship of atrial function with cardiac function in the late phase more than 20 years after a Fontan operation

**DOI:** 10.1093/icvts/ivac066

**Published:** 2022-03-18

**Authors:** Tomomitsu Kanaya, Masaki Taira, Takayoshi Ueno

**Affiliations:** Department of Cardiovascular Surgery, Graduate School of Medicine, Osaka University, Osaka, Japan

**Keywords:** Atrial function, Fontan, cardiac index, cardiac computed tomography

## Abstract

**OBJECTIVES:**

Atrial function in Fontan patients is unknown. Our goal was to report the relationship of atrial function with the cardiac index and atrial function and clinical outcome through longer follow-up periods.

**METHODS:**

Twelve patients were followed up for over 20 years after their first Fontan operation. Atrial function, including the expansion index, atrial ejection fraction, passive ejection fraction and active ejection fraction, was examined using cardiac computed tomography. The relationship of atrial function with the cardiac index and failing Fontan patients was analysed.

**RESULTS:**

Twelve Fontan patients were included. The median follow-up period after the first Fontan operation was 27 (range, 21-33) years, and the median age of those examined was 33.5 (range, 24-60) years. There were 6 male patients (50%). The cardiac index showed a significant positive correlation with the expansion index (*P* = 0.02), the atrial ejection fraction (*P* = 0.035), and the active ejection fraction (*P* = 0.013). The expansion index (39.2 ± 19.6 vs 64.1 ± 3.9), atrial ejection fraction (26.6 ± 10.9 vs 39.0 ± 1.5%), booster pump (15.6 ± 9.0 vs 31.3 ± 3.5) and cardiac index (2.1 ± 0.3 vs 2.5 ± 0.2 L/min/m^2^) were significantly lower in patients with a history of arrhythmia than in patients without a history of arrhythmia (*P* < 0.05). The expansion index (23.5 ± 13.5 vs 59.5 ± 8.7), atrial ejection fraction (18.1 ± 8.6 vs 37.1 ± 3.7) and active ejection fraction (7.3 ± 2.7 vs 27.7 ± 5.2) were significantly lower in failing Fontan patients than in non-failing Fontan patients (*P* < 0.01).

**CONCLUSIONS:**

Patients with atrial arrhythmia and signs of Fontan failure have lower atrial function than those without.

**Figure:**
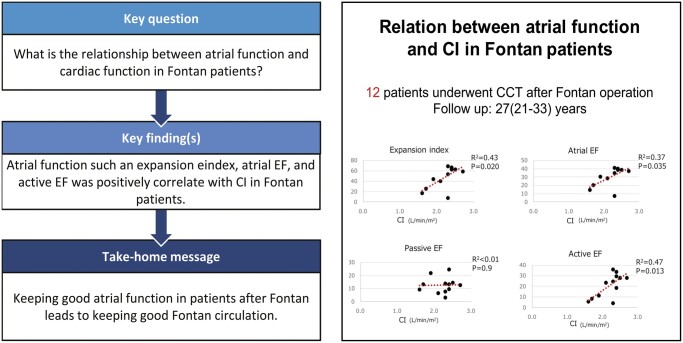

## INTRODUCTION

The Fontan operation, first reported by Fontan in 1971 for the treatment of tricuspid atresia [1], is currently the standard procedure for patients with single ventricular physiology. In the late phase, some patients have a failing Fontan and low cardiac function [2–4]. The causes of decreasing cardiac function vary; however, the atrial function in Fontan circulation is not fully elucidated [5, 6]. The relationship between atrial function and overall cardiac function is well known in normal biventricular systems, and it is understood that atrial kick contributes to 20% to 30% of the cardiac output and plays an important role in good cardiac circulation [7, 8].

Atrial tachycardia increases in the late phase of the Fontan procedure [9] Atrial tachycardia results in dilatation of the atrium and fibrosis of the atrium. It is also reported that the cardiac index after the Fontan procedure decreases with time [10] and that the number of failed Fontan patients also increases. Ven *et al.* [5] clarified the role of the atrium for Fontan patients with an extracardiac conduit. However, its role and influence among the failed Fontan patients are not well understood. The goal of this study was to reveal the relationship of atrial function with haemodynamics and clinical outcome in patients with arrhythmia and in failed Fontan patients.

## MATERIALS AND METHODS

### Ethical statement

The Ethics Committee of the Osaka University Hospital approved the design of this study; written informed consent was obtained from all patients. This retrospective study was approved by our institutional review board on 2 November 2016 (approval number 16105). All patients

### Patients

This retrospective study included 12 patients who had been followed up at the Osaka University Hospital for > 20 years since their first Fontan operation and whose atrial function could be analysed with cardiac computed tomography (CCT) while maintaining sinus rhythm or atrial all-pacing rhythm.

Demographic and clinical data were obtained from a review of the medical records, including cardiac anatomy, Fontan type, age at Fontan operation, time from Fontan to CCT, history of arrhythmia and cardiac function obtained from cardiac catheterization. Patient characteristics are shown in Table 1.

**Table 1:** Patient characteristics

	n = 12
Age, (years)	33.5 (24–60)
Male, n (%)	6 (50)
Age at Fontan, n (years)	5 (5-37)
Follow-up periods after Fontan, n (years)	27 (21-33)
Periods from Fontan to CT, (years)	24.5(14.4-29.7)
Periods from Fontan to cardiac catheterization, (years)	23.8(16.0-30.8)
Periods between CT and cardiac catheterization, (years)	1.34(0-4.6)
Underlying disease, n (%)	
Tricuspid atresia	3 (25)
DILV	2 (16)
Unbalanced AVSD	2 (16)
Mitral atresia	1 (8)
VSD	1 (8)
DORV	1 (8)
PA/IVS	1 (8)
Other	1 (8)
First Fontan type, n (%)	
Atriopulmonary connection	6 (50)
Lateral tunnel	4 (33)
Extracardiac	2 (16)
Definitive Fontan type, n (%)	
Lateral tunnel	2 (16)
Extracardiac	10 (83)

AVSD: atrioventricular septal defect; CT: computed tomography; DILV: double inlet left ventricle; DORV: double outlet right ventricle; PA/IVS: pulmonary atresia with intact ventricular septum VSD: ventricular septal defect.

In all patients, we examined the catheter-related parameters such as cardiac index (CI), cardiac central venous pressure, end-diastolic pressure, ejection fraction (EF), pulmonary artery index, pulmonary vascular resistance and each atrial function.

After a detailed review of the medical records, patients with a history of arrhythmia treatment, including a pacemaker implant, the MAZE procedure and radiofrequency catheter ablation were classified as group A (*n* = 8) and patients with no history of treatment were classified as group NA (*n* = 4). The cardiac indices of both groups were compared.

Clinical Fontan failure was defined as death, a collection of pleural effusion or ascites and a New York Heart Association functional classification III or above (group F). Cases without such a history were defined as the non-failing Fontan group (group NF). Failed Fontan patients were as follows: 1 patient underwent an atriopulmonary connection (APC) Fontan procedure at the age of 7 years and subsequently an extra-cardiac total cavopulmonary connection (EC-TCPC) at the age of 20 years. She suffered from chronic renal failure, ascites collection and hypoalbuminemia. Pleural effusion and ascites collection were repeatedly collected, and she died at the age of 37 years. One patient underwent the APC Fontan procedure at the age of 38 years and subsequently an EC-TCPC conversion at the age of 52 years. She had chronic renal failure with 2.0 to 3.0 mg/dl of serum creatinine. She died at the age of 63 years of renal failure. These 2 patients died 6 and 12 years after the computed tomography (CT) scan, respectively, and 3 and 6 years after the MRI. One patient, by the time of this study, has chronic ascites collection; as much as 2000 ml every month was drained. One patient had no ascites and pleural effusion, but her symptoms of fatigue and dyspnoea on exertion were severe. She had been hospitalized and discharged repeatedly due to her symptoms.

### Cardiac computed tomography

Postoperative atrial volume was evaluated using CCT. Multidetector cardiac CT examinations were performed using a 64-slice CT system (Discovery CT750 HD; GE Healthcare, Milwaukee, WI, USA) and an iodinated contrast agent (iopamiron 370; Bayer Healthcare, Tokyo, Japan). After the reformatted images were transferred to a workstation, contiguous multiphase short-axis images were generated using semi-automated interactive software (Advantage Workstation 4.6, CardIQ Xpress function; GE Healthcare, Buckinghamshire, UK) [11]. The atrium was defined as the atrium connected to the systemic chamber and a single atrium or a combination of the right and left atria in which the lateral tunnel was excluded from pulmonary venous volumes, whereas atrial appendages were included and measured by CCT.

### Atrial function

The atrial volume analysis during the entire cardiac cycle length describes atrial functions. The atrium receives blood from the pulmonary veins, increasing its volume from minimum to maximum during the ventricular systole. The atrial endocardial borders were detected by visual inspection using volume rendering and multiplanar two-dimensional (2D) images. We drew a 2D trace of images in 2 directions (coronal and sagittal) and converted the morphology of the atrium into a three-dimensional (3D) image. One radiologist and one cardiac surgeon checked the 2D and 3D images so that the 3D image accurately reflected the atrial volume. The average value of the atrial volume measured for each was adopted as the atrial volume. The atrial volume connected to the systemic chamber was calculated. If the patients have had an intra-atrial conduit, the volume of the conduit was removed. This atrial volume was calculated to be that of the left atrium. The atrial volume was calculated every 10% of the percentage of the RR interval, which was analysed in 1 cardiac cycle. A cycle of the maximum, the minimum and atrial pre-atrial contraction volumes was drawn and calculated.

This function is affected by the atrial reservoir function (expansion index) and the atrial global function (atrial EF). The conduit function is influenced by atrial compliance as well as by the passive ejection fraction. The booster pump actively augments the ventricular filling as an active EF during late ventricular diastole [12, 13]. The patients’ basic atrial functions are shown in Table 2.

**Table 2:** Volumetric indexes of atrial function

Index	Atrial function	Calculation	n = 12
Expansion index	Atrial reservoir function	(A_max_-A_min_)/A _min_	55.9 (7.5-68.8)
Atrial ejection fraction	Atrial global function	(A_max_-A_min_)/A_max_	35.8 (6.9-40.7)
Passive ejection fraction	Conduit function	(A_max_-A_preA_)/A_max_	13.0 (3.1-24.6)
Active ejection fraction	Booster pump	(A_preA_-A_min_)/A_preA_	23.9 (4.0-35.8)

A_max_: maximal atrial volume; A_min_: minimal atrial volume; A_preA_: atrial volume immediately before atrial contraction.

### Statistical analyses

Continuous variables are presented as median (maximum to minimum) or mean ± standard deviation. Categorical variables are reported as frequencies. Intergroup comparisons were performed using an unpaired Student *t*-test. Regression analysis was performed to determine the correlation between the Fontan circulation and atrial function using a univariable model with just 2 factors. All statistical analyses were performed using JMP Pro 14 software (SAS Inc., Cary, NC, USA), and a *P*-value of <0.05, which was considered statistically significant.

## RESULTS

**Figure 1:**
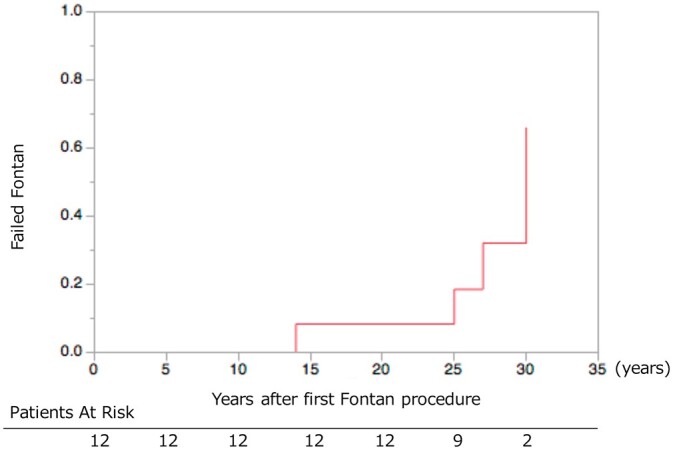
Rate of failed Fontan patients at follow-up.

Twelve patients with Fontan were included in this study (Table 1). They underwent the first Fontan operation between 1983 and 1991. The median age was 33.5 (range, 24–60) years. Age at the first Fontan operation age was 5 (range, 5–37) years; the follow-up period from the initial Fontan operation was 27 (range, 21–33) years and the male-female ratio was 1:1. APC as the first Fontan type was present in 6 (50%) patients; the lateral tunnel was present in 4 (33%) patients and the extracardiac conduit was present in 2 patients (16%). The definitive Fontan type was the lateral tunnel (16%) and the extracardiac conduit (83%). None of the patients had fenestration. The failed Fontan curve is shown in Fig. 1. The variables of cardiac and pulmonary functions are shown in Table 3.

**Table 3:** Variables of cardiac and pulmonary functions

Variables	n = 12
Heart rate, (bpm)	79.5 (68-102)
Sinus rhythm, n	8
Atrial pacing rhythm, n	4
CI (L/min/m^2^)	2.3 (1.6-2.7)
CVP, (mmHg)	14 (9-18)
EF, (%)	46 (26-63)
EDP, (mmHg)	10 (7-14)
PAI	153 (85-536)
PVR, (U)	1.6 (0.6-4.0)

bpm: beats/minute; CI: cardiac index; CVP: central venous pressure; EDP: end-diastolic pressure; EF: ejection fraction; PAI: pulmonary arterial index; PVR: pulmonary vascular resistance.

Basic haemodynamic parameters, including catheterization data and atrial function, are shown in Table 4. In group A, 5 patients (63%) underwent PMI, 5 patients (63%) underwent the Maze procedure and 1 patient (13%) underwent radiofrequency catheter ablation .

**Table 4:** Comparison of the basic characters and cardiac parameters with group A and group NA

	Group A	Group NA	*P*-value
	n = 8	n = 4	
Age (years)	37.5 (31-63)	32.5 (26-35)	0.11
Age at first Fontan operation, (years)	6 (5-37)	5 (5-10)	0.47
Duration from first Fontan operation, (years)	29 (22-35)	26 (21-28)	0.20
Male, n (%)	1 (8)	3 (75)	0.03
First Fontan type, n (%)			0.06
APC	6 (73)	1 (25)	
LT-TCPC	2 (25)	1 (25)	
EC-TCPC	0	2 (50)	
Procedure for arrhythmia, n (%)			
PMI	5 (42)	0	0.02
Maze procedure	4 (33)	0	0.02
RFCA	1 (8)	0	0.18
Deaths, n (%)	2 (17)	0	0.18
Catheter data			
Heart rate, (bpm)	80.3 ± 10.9	79.8 ± 11.2	0.94
Sinus rhythm, n	3 (25)	4 (33)	0.09
Atrial pacing rhythm, n	5 (42)	0	0.02
CI, (L/min/m^2^)	2.1 ± 0.3	2.5 ± 0.1	0.05
CVP, (mmHg)	13.6 ± 2.8	14.0 ± 1.4	0.82
EF, (%)	44.1 ± 11.5	54.0 ± 8.4	0.19
EDP, (mmHg)	10.5 ± 2.2	10.5 ± 0.9	1.0
PAI	242.2 ± 166.6	172.3 ± 35.1	0.47
PVR, (U)	2.1 ± 1.2	1.4 ± 0.3	0.30
Atrial function			
Expansion index	39.2 ± 19.6	64.6 ± 3.9	0.04
Atrial EF	26.6 ± 10.9	39.2 ± 1.5	0.07
Passive EF	13.2 ± 6.8	11.1 ± 2.6	0.59
Active EF	15.6 ± 9.0	30.8 ± 3.5	0.01

Group A: Patients with a history of treatment of arrhythmia.

Group NA: Patients with no history of treatment of arrhythmia.

APC: atriopulmonary connection; CI: cardiac index; CVP: central venous pressure; EC-TCPC: extra cardiac–total cavopulmonary connection; EDP: end-diastolic pressure; EF: ejection fraction; LT-TCPC: lateral tunnel-total cavopulmonary connection; PAI: pulmonary artery index; PMI: pacemaker implant; PVR: pulmonary vascular resistance; RFCA: radiofrequency catheter ablation.

### Correlation between the cardiac index, other measurements of cardiac catheterization and atrial function

**Figure 2:**
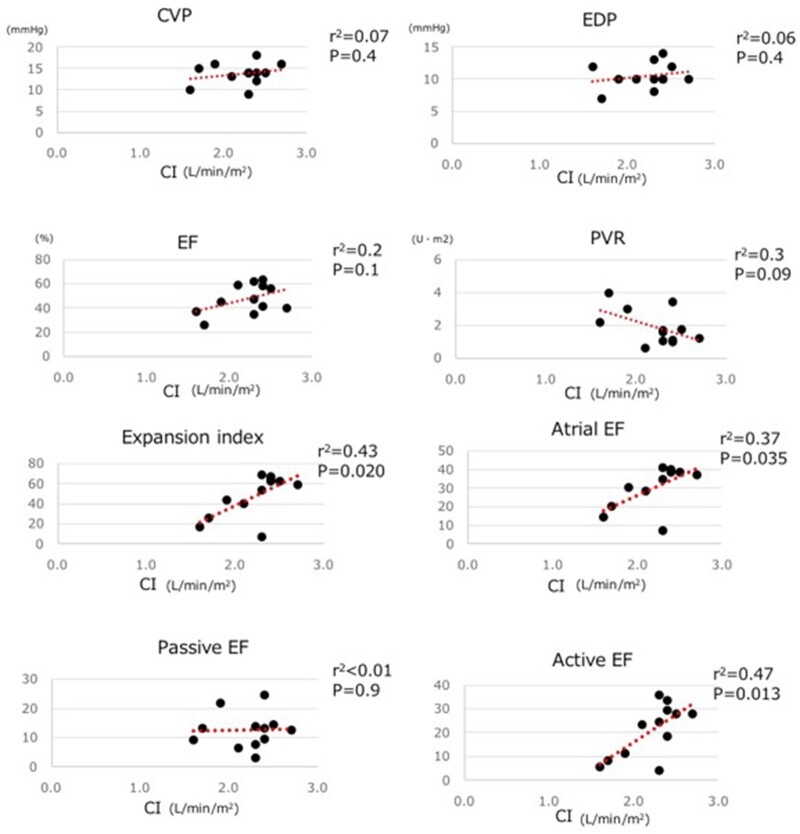
Relationship of cardiac index with cardiac index and cardiac catheter results and atrial function.

The CI was not significantly correlated with other cardiac functions such as central venous pressure, end-diastolic pressure, EF, the pulmonary artery index and pulmonary vascular resistance (Fig. 2). However, the CI showed a significant positive correlation with the expansion index (*P* = 0.02), the atrial EF (*P* = 0.04) and the active EF (*P* = 0.01).

### Comparison of atrial function and cardiac index in the arrhythmia group (group A) and the non-arrhythmia group (group NA)

**Figure 3:**
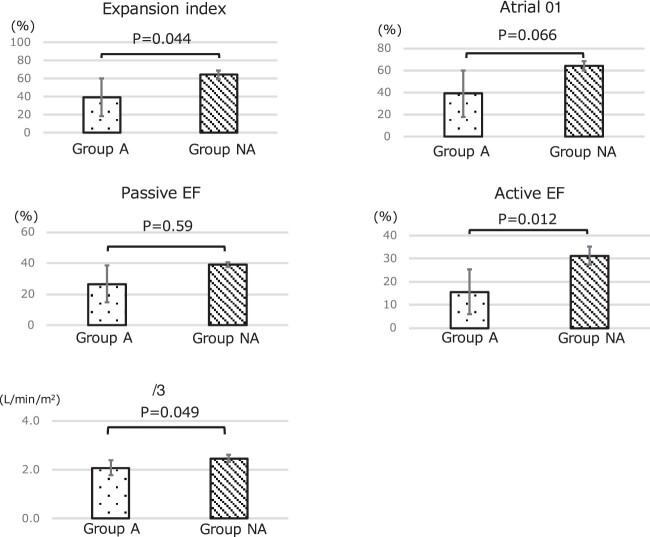
Comparison of each atrial function and cardiac index between the patients with a history of arrhythmia (group A) and those without that (group NA).

Atrial function was evaluated using the expansion index, atrial EF, the booster pump and conduit function. Each atrial function was compared between groups A and NA (Table 4). The expansion index (39.2; 95% confidence interval, 25.3–53.2 vs 64.1; 95% confidence interval, 44.4-83.9; *P* = 0.044) and active EF (15.6; 95% confidence interval, 9.1-22.2 vs 31.3; 95% confidence interval, 22.0-40.6; *P* = 0.01) were significantly lower in group A than in the remaining groups. The CI was also significantly lower in group A (2.1; 95% confidence interval, 1.8-2.3 vs 2.5; 95% confidence interval, 2.2–2.8; *P* = 0.049) (Fig. 3). Group A had a lower atrial function and lower CI than group NA.

### Comparisons of atrial function between patients with a failing Fontan and a non-failing Fontan

The atrial functions between the failing Fontan group (group F) and the non-failing Fontan group (group NF) were compared (Table 5). The expansion index (23.5; 95% confidence interval, 10.6–36.4 vs 59.5; 95% confidence interval, 50.4-68.6; *P* = 0.001), atrial EF (18.1; 95% confidence interval, 11.0-25.2 vs 37.1; 95% confidence interval, 32.1-42.1; *P* = 0.001) and active EF (7.3; 95% confidence interval, 1.7-12.8 vs 27.7; 95% confidence interval, 23.7-31.6; *P* = 0.0001) were significantly lower in group F than in group NF. There were no significant differences in the remaining parameters between the groups, except for the CI (1.9; 95% confidence interval, 1.6-2.1 vs 2.4; 95% confidence interval, 2.2–2.5; *P* = 0.004) (Table 5).

**Table 5:** Comparison of the basic characteristics and cardiac parameters between group F and group NF

	Group F	Group NF	*P* value
	n = 8	n = 4	
Age (years)	33 (26-35)	44.5 (40-63)	<0.01
Age at first Fontan operation, (years)	5 (4-10)	10.5 (7-37)	0.09
Duration from first Fontan operation, (years)	26.5 (21-30)	33 (22-35)	0.05
Male, n (%)	4 (33)	0	0.1
First Fontan type, n (%)			0.2
APC	4 (33)	4 (33)	
LT-TCPC	2 (17)	0	
EC-TCPC	2 (17)	0	
Procedure for arrhythmia, n (%)			
PMI	2 (17)	3 (25)	0.07
Maze procedure	2 (17)	2 (17)	0.39
RFCA	1 (8)	0	0.35
Mortality, n (%)	2 (17)	0	
Catheter data			
Heart rate, (bpm)	80.1 ± 12.4	80.0 ± 3.2	0.99
Sinus rhythm, n	1 (8)	6 (50)	0.09
Atrial pacing rhythm, n	3 (25)	2 (17)	0.09
CI, (L/min/m^2^), average	2.4 ± 0.2	1.9 ± 0.3	0.004
CVP, (mmHg), average	13.8 ± 2.5	13.8 ± 2.3	1.0
EF, (%), average	51.8 ± 10.5	38.8 ± 8.3	0.08
EDP, (mmHg), average	10.5 ± 1.7	10.5 ± 2.3	1.0
PAI. average	201.5 ± 104.0	258.0 ± 198.5	0.59
PVR, (U), average	1.5 ± 0.8	2.7 ± 0.9	0.06
Atrial function			
Expansion index, average	59.5 ± 8.7	23.5 ± 13.5	0.001
Atrial EF, average	37.1 ± 3.7	18.1 ± 8.6	0.001
Passive EF, average	12.8 ± 5.2	11.9 ± 6.8	0.82
Active EF, average	27.7 ± 5.2	7.3 ± 2.7	<0.0001

APC: atriopulmonary connection; CI: cardiac index; CVP: central venous pressure; EC-TCPC: extracardiac-total cavopulmonary connection; EDP: end-diastolic pressure; EF: ejection fraction; group F: failed Fontan group; group NF: non-failed Fontan group; LT-TCPC: lateral tunnel-total cavopulmonary connection; PAI: pulmonary artery index; PMI: pacemaker implant; PVR: pulmonary vascular resistance; RFCA: radiofrequency catheter ablation.

## DISCUSSION

The results of this study have indicated that the atrial function measured using CCT correlated positively with the CI by invasive catheter examination and clinical symptoms. Atrial function, such as the expansion index, atrial EF, passive EF and active EF, were expected to be an important indicator of Fontan circulation.

Peck *et al.* demonstrated a correlation between atrial strain variables by echocardiography with CI and their importance for Fontan circulation [14]. This study also showed the correlation of atrial function with CI and Fontan failure. Patients with arrhythmia and a history of treatment had low atrial function and a low CI. In the treatment of a single ventricle, APC or a lateral conduit TCPC, which requires atriotomy and load to the atrium, may lead to an increase in the substrate of the arrhythmia and the occurrence of sinus node dysfunction, resulting in low atrial function. Van *et al.* demonstrated that patients with EC-TCPC had higher atrial reservoir function than patients with LT-TCPC, but atrial function did not predict exercise capacity or events [7]. In this study, a few patients had EC-TCPC, and many patients underwent EC-TCPC conversion from APC or LT-TCPC; therefore, the superiority of EC-TCPC remains unknown. However, 6 (73%) members of group A had an APC as their first Fontan type of operation. The members of group A had a lower atrial function and lower CI than those in group NA. All APC Fontan patients except for 1 were included in group A. But their atrium was still large and their atrial function was very low in the late periods. It was possible that the atrium of the APC Fontan has been exposed to Fontan pressure for a long time and is under stress, resulting in atrial damage. We would like to propose that a procedure that receives atrial volume and pressure load may be considered to have harmful effects on the late Fontan circulation. Regarding the maintenance of atrial function, the selection of a surgical method without atriotomy and volume load to the atrium is important. Heiner [15] reported that the surgical incisions at the atria and the presence of atrioventricular valve regurgitation might have harmful effects on the fragile Fontan circulation. In our cohort, the patient with very low atrial function due to severe atrioventricular regurgitation died 30 years after the Fontan operation. The patient had a huge atrial volume, low systolic and diastolic function and low atrial function. This study did not analyse the influence of atrioventricular valve regurgitation on atrial function; however, to protect the atrial function in the long term, we suggest that atrial function be examined before deciding the timing of the operation for atrioventricular regurgitation.

Ven *et al.* [5] showed that patients with extracardiac Fontan have high reservoir function and low conduit function. However, what atrial function indicated for a failed Fontan was unknown until now. In this study, the authors found that the atrial function, including reservoir function and pump function, of patients with a failed Fontan was reduced. However, since the CI of the failed Fontan is low, and the CI and the atrial function are related, it was considered that maintaining atrial function may be one of the factors for maintaining the Fontan circulation. To maintain atrioventricular function, it is important to avoid atrioventricular valve regurgitation, which is a volume load. The APC procedure [16], which is also an atrial volume load, has to be avoided. To maintain Fontan circulation in the late period, it is important to consider controlling atrioventricular valve regurgitation and the selection of a Fontan type to ensure that atrial function does not deteriorate.

## LIMITATIONS

This study has several limitations. It was a retrospective, single-centre study that included a small number of patients. First, the statistical analysis may not have had sufficient power to permit drawing firm conclusions. In a heterogeneous population with such small numbers, significant caution needs to be exercised when interpreting the data. These patient data were small in number but less variable and tended to be shown. We performed this study as a pilot study to explore the relationship between atrial function and failing Fontan circulation. Second, the evaluation of atrial function and volumetry of both atriums or a single atrium is performed by echocardiography and MRI as reported in references [7, 8, 13]. Some studies have evaluated atrial function in the right atrium [12], but such a study of Fontan patients, to the best of our knowledge, does not exist, and no validation study was reported. Fontan circulation and failure can be affected by various haemodynamic and circulatory factors beyond CI and non-cardiovascular factors, which were not fully evaluated in this study. The anatomy of the atria/atrium is usually extremely complex after the Fontan pathway and poor contrast opacification is possible. In our study, whether it had atria or an atrium, the atrial volume was calculated by approximating the left atrium. Therefore, it may be possible that it does not reflect accurate atrial volume and function.

## CONCLUSIONS

Fontan circulation, clinical outcomes and atrial function tend to be closely related. In this study, patients with atrial arrhythmia and signs of Fontan failure have lower atrial function. The implications of these findings need to be evaluated in a longitudinal manner to assess the impact on Fontan failure.

## ABBREVIATIONS AND ACRONYMS

CCT: Cardiac computed tomography
APC: Atriopulmonary connection
EC-TCPC: Extracardiac total cavopulmonary connection
CI: Cardiac index
EF: Ejection fraction
